# LiT: limit order book transformer

**DOI:** 10.3389/frai.2025.1616485

**Published:** 2025-10-13

**Authors:** Yue Xiao, Carmine Ventre, Yuhan Wang, Haochen Li, Yuxi Huan, Buhong Liu

**Affiliations:** ^1^Finance Hub, Department of Informatics, King's College London, London, United Kingdom; ^2^School of Computing and Mathematical Sciences, Birkbeck, University of London, London, United Kingdom; ^3^Department of Computer Science, University College London, London, United Kingdom; ^4^School of Finance and Management, SOAS University of London, London, United Kingdom

**Keywords:** transformers, deep learning, limit order book, high-frequency trading, market microstructure, representation learning, transfer learning

## Abstract

While the transformer architecture has demonstrated strong success in natural language processing and computer vision, its application to limit order book forecasting, particularly in capturing spatial and temporal dependencies, remains limited. In this work, we introduce Limit Order Book Transformer (LiT), a novel deep learning architecture for forecasting short-term market movements using high-frequency limit order book data. Unlike previous approaches that rely on convolutional layers, LiT leverages structured patches and transformer-based self-attention to model spatial and temporal features in market microstructure dynamics. We evaluate LiT on multiple LOB datasets across different prediction horizons, LiT consistently outperforms traditional machine learning methods and state-of-the-art deep learning baselines. Furthermore, we show that LiT maintains robust performance under distributional shifts via fine-tuning, making it a practical solution for fast-paced and dynamic financial environments.

## 1 Introduction

In the context of the rapid shift toward automated trading in modern financial markets, the Limit Order Book (LOB) has emerged as a central focus for studying market microstructure. The LOB is a centralized system that records buy and sell orders submitted by market participants, aggregating order volumes at discrete price levels. With the rapid generation of high-frequency LOB data along with the advancement in machine learning models and computational resources, forecasting short-term market movements has become feasible and increasingly valuable for supporting decision-making in fast-paced trading environments. However, the complex structure of the limit order book, characterized by its latent dynamics and deep hierarchy, makes LOB feature representation and extraction particularly challenging. Moreover, the high volatile, noisy and non-stationary nature of LOB data further complicates the market trend prediction.

Traditional market forecasting models relied on hand-crafted features and statistical methods, but these approaches have proven insufficient for capturing the complex dynamics and nonlinear patterns in real-world high-frequency LOB data. The past decade has seen increasing adoption of machine learning techniques, especially deep learning approaches that automatically learn feature representation from raw LOB inputs. Among these approaches, Convolutional Neural Networks (CNNs; LeCun et al., 1998) have become particularly dominant. Pioneering works such as DeepLOB (Zhang et al., 2019) showed that CNNs, when combined with models like Long Short-Term Memory (LSTM; Hochreiter and Schmidhuber, 1997) to capture temporal dependencies, can effectively model spatial and temporal dependencies in LOBs and achieve state-of-the-art performance. More recently, based on the transformer architecture (Vaswani et al., 2017), approaches such as TransLOB (Wallbridge, 2020) have demonstrated that transformers, when combined with CNNs for feature extraction, can also effectively model market dynamics in LOB data.

Building on these developments, we propose the Limit Order Book Transformer (LiT), a transformer-based architecture that eliminates the need for convolutional layers in LOB forecasting. Inspired by the success of transformer models in Natural Language Processing (NLP) and Computer Vision (CV), LiT captures LOB features using structured patches and processes them through self-attention layers, followed by LSTM layers to enhance temporal modeling. Unlike prior approaches, LiT combines the expressive power of transformers with the sequential modeling capabilities of recurrent networks, enabling it to efficiently learn both spatial and temporal features in LOB across short and long term dependencies, as well as maintaining a capability to adapt to the latest market conditions.

Our main contributions are as follows:

- We propose LiT, a novel transformer-based model for LOB forecasting that replaces convolutional layers with a structured patch-based self-attention mechanism.
- We benchmark LiT against traditional ML models, deep learning baselines and state-of-the-art CNN-based architectures across multiple prediction horizons and show consistent improvements.
- We conduct a comprehensive analysis of structured patch configurations in LiT and show that narrower temporal windows and deeper spatial coverage significantly improve LiT forecasting performance.
- We show that LiT remains strong performance under distributional shift in market dynamics via fine-tuning, making it practical for real-world deployment.

The remainder of the paper is organized as follows. Section 2 reviews related work in LOB forecasting. Section 3 describes the data collection and preparation. Section 4 outlines our proposed architecture and training methodology. Section 5 presents experimental results, including a comparison across models, patch size analysis, and time-adaptive fine-tuning. Finally, Section 6 concludes the paper and discusses future research directions.

## 2 Related work

### 2.1 Statistical methods and traditional ML techniques

Early adoption of statistical methods and traditional machine learning approaches for analyzing limit order book data typically emphasizes simplicity and interpretability. Statistical approaches such as Autoregressive Integrated Moving Average (ARIMA), Vector Autoregressive models (VAR) and Generalized Autoregressive Conditional Heteroskedasticity (GARCH) primarily explore linear relationships between LOB-derived signals and target variables such as price and volatility. For example, Ariyo et al. (2014) employed ARIMA for short-term stock price prediction, while Pai and Lin (2005) enhanced stock forecasting by integrating ARIMA with Support Vector Machines (SVM) to capture nonlinear patterns. Traditional machine learning methods, including regression models, SVMs, and random forests, have also been applied to capture more complex dynamics inherent in market microstructures, especially when statistical assumptions like linearity and stationarity are not met. Zheng et al. (2012) leveraged LASSO logistic regression for feature selection to predict price jumps, while Krauss et al. (2017) adopted gradient boosting and random forests in an ensemble framework for statistical arbitrage on the S&P 500 index. Additionally, Kercheval and Zhang (2015) and Li et al. (2016) applied SVMs to predict market movements by categorizing them into different trends based on predefined thresholds.

### 2.2 Conventional deep learning approaches

With developments in deep learning and computational resources in recent decades, deep learning has become a mainstream approach in limit order book research. Different neural network architectures have been extensively explored in numerous studies. Basic models like Multilayer Perceptrons (MLP) are usually employed as benchmark models. For instance, Ntakaris et al. (2018) created a LOB dataset and applied a shallow neural network for market movement forecasting. The LSTMs (Hochreiter and Schmidhuber, 1997) are commonly employed due to their effectiveness in capturing long-term temporal dependencies, Sirignano and Cont (2019) demonstrated improved performance by training an LSTM model on multiple stocks compared to a single-stock model. Fang et al. (2021) adopted a two-layer LSTM model to predict market movements and evaluate its performance over time. Meanwhile, CNNs have been frequently applied due to their effectiveness in extracting spatial features from grid-like LOB data. For example, CNNs have been shown to outperform MLP and SVM models in predicting market movements (Tsantekidis et al., 2017). Furthermore, hybrid approaches combining LSTM and CNN architectures have also been explored, as illustrated by Tsantekidis et al. (2020), and further popularized by Zhang et al. (2019), becoming state-of-the-art benchmarks.

### 2.3 Advanced transformer-based models

Following the invention of attention mechanisms (Bahdanau, 2014) and transformers (Vaswani et al., 2017), transformer-based models have greatly advanced in various fields, especially NLP and CV. In recent years, transformer models have also attracted research in the financial domain, particularly in limit order book (LOB) forecasting. Wallbridge (2020) combined CNNs with transformers to predict LOB movements, while Sridhar and Sanagavarapu (2021) applied attention mechanisms to forecast cryptocurrency price movements. Zhang et al. (2021) applied deep learning to Market-by-Order (MBO) data for high-frequency forecasting, showing its complementary value to LOB-based models. More recently, Arroyo et al. (2024) introduced a convolutional-transformer model to estimate fill probabilities in the LOB using survival analysis. While their architecture is similar in structure, their focus is on order execution timing rather than market movement forecasting, making their work complementary to ours.

However, CNN-based approaches encounter limitations due to spatial inductive biases, which do not align effectively with intrinsic LOB characteristics. Typically, LOB features exhibit hierarchical properties, where levels near the mid-price update more frequently than deeper levels, thus reducing the utility of spatial locality assumptions inherent in CNNs. In contrast, this paper proposes a sophisticated model architecture that completely removes CNN reliance. We demonstrate that eliminating CNN components does not compromise predictive performance, thereby confirming the efficacy and adaptability of transformer-based methods for modeling complex LOB dynamics.

## 3 Limit order book data

### 3.1 Limit order book overview

A limit order book is a centralized record that facilitates the matching of buy and sell orders submitted by market participants. The LOB aggregates limit orders, which are orders that wait for a desired price to be reached rather than execute immediately with certainty at the current market price, on both the sell side and the buy side of the book. Each buy or sell order is placed with a specific quantity at a specified price, and multiple orders at the same price level are aggregated in the LOB. On the sell side, participants seek to sell assets, and their orders are ranked from lowest to highest price, with the lowest ask price given the highest execution priority. Conversely, buy orders are ranked from highest to lowest bid price. Incoming market orders are matched against the best available limit orders. The order matching process may follow different market rules, such as price-time priority (also known as First-in-First-out) or pro-rata matching, depending on the policy of the platform.

**Definition** A *limit order book* (LOB) with *n* levels of price and volume is defined as the set


$$X=\left\{x_{1},x_{2},\ldots ,x_{n}\right\},$$

where each level *x*_*i*_ is a tuple


$$x_{i}=\left(P_{i}^{\text{ask}},V_{i}^{\text{ask}},P_{i}^{\text{bid}},V_{i}^{\text{bid}}\right),\;\text{for }i=1,2,\ldots ,n.$$

Here, $P_{i}^{\text{ask}}$ and $V_{i}^{\text{ask}}$ denote the ask price and volume at level *i*, while $P_{i}^{\text{bid}}$ and $V_{i}^{\text{bid}}$ represent the bid price and volume at the same level. The *best ask price* $P_{1}^{\text{ask}}$ (i.e., the lowest sell price) and the *best bid price* $P_{1}^{\text{bid}}$ (i.e., the highest buy price) are defined as:


$$P_{1}^{\text{ask}}=\underset{i}{\min}P_{i}^{\text{ask}},\;P_{1}^{\text{bid}}=\underset{i}{\max}P_{i}^{\text{bid}}.$$

The *mid-price* at time *t* is given by:


$$p_{\text{mid}}^{t}=\frac{P_{1}^{\text{ask}}+P_{1}^{\text{bid}}}{2}.$$

**Figure 1:**
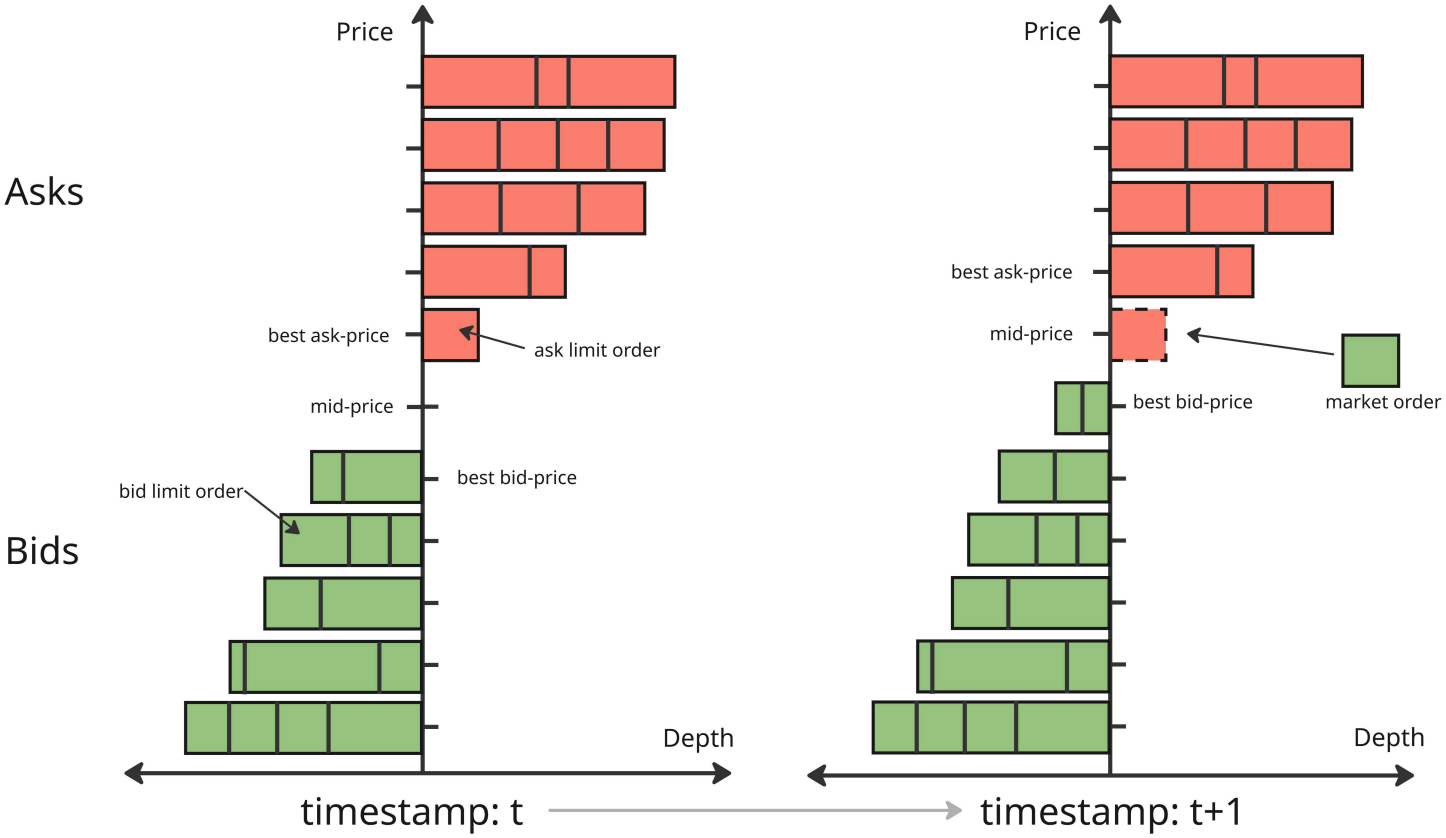
Limit order book evolution from time *t* to *t*+1.

A market movement from time *t* to time *t*+1 is illustrated in Figure 1. The horizontal axis represents the market depth at each price level, while the vertical axis represents the price levels. In the figure, red bars indicate sell orders and green bars correspond to buy orders, with the block sizes representing order volumes. At time *t*, there are five price levels on both the bid and ask sides. At time *t*+1, the ask limit order at the best ask price $P_{1}^{\text{ask}}$ is matched with an incoming market buy order, leading to its removal from the order book. As a result, the best ask price moves up to the next available level, and the mid-price $p_{\text{mid}}^{t}$ shifts accordingly to reflect the new best bid and ask prices. In our experiments, we use the mid-price as a proxy for market movement and evaluate the predictive performance of various models based on its changes over time.

### 3.2 Data collection and preparation

To evaluate our proposed model architecture, we collect Level 2 high-frequency book data from the Binance exchange. The full order book is reconstructed at the millisecond level, and we use the top 20 levels on both the bid and ask sides as input to our model. This leads to 80 features of price and volume information at each timestamp, capturing the market depth on both sides.

We collect four datasets to support the different experimental setups discussed in Section 5: one covering the full month of September 2024, and three others consisting of data from the second week of each month in October, November, and December. Since the cryptocurrency market operates 24 h a day and the sampling interval between timestamps is extremely small, the resulting dataset is significantly more granular than conventional daily price data. We consider this data volume sufficient to support robust evaluation and fair performance comparison across different models. Following the event-based inflow approach adopted in Ntakaris et al. (2018), we construct the LOB dataset with price and aggregated volume information at each price level for both bids and asks. In total, over 1 million LOB snapshots are reconstructed from the streaming data. The descriptive statistics of the datasets are presented in Table 1. The mid-price distributions across all months show low skewness and mostly negative kurtosis, indicating relatively symmetric distributions with thinner tails than a normal distribution. These characteristics suggest that extreme price movements are infrequent, reducing the risk of extreme outliers significantly impacting model performance.

**Table 1:** Descriptive statistics of LOB datasets across different months.

	**Timestamps**	**Mean**	**St. Dev**	**Min**	**Max**	**Skew**	**Kurtosis**
***September***	1,077,057	59,038.824	3,206.560	52,550.005	66,076.115	0.284	−1.110
***October***	177,651	70,149.906	2,104.124	66,439.905	73,620.115	−0.235	−1.477
***November***	130,246	69,223.755	807.258	67,478.735	71,632.805	0.511	0.261
***December***	233,058	95,574.438	2,135.029	91,532.525	99,963.695	0.256	−1.087

## 4 Method

**Figure 2:**
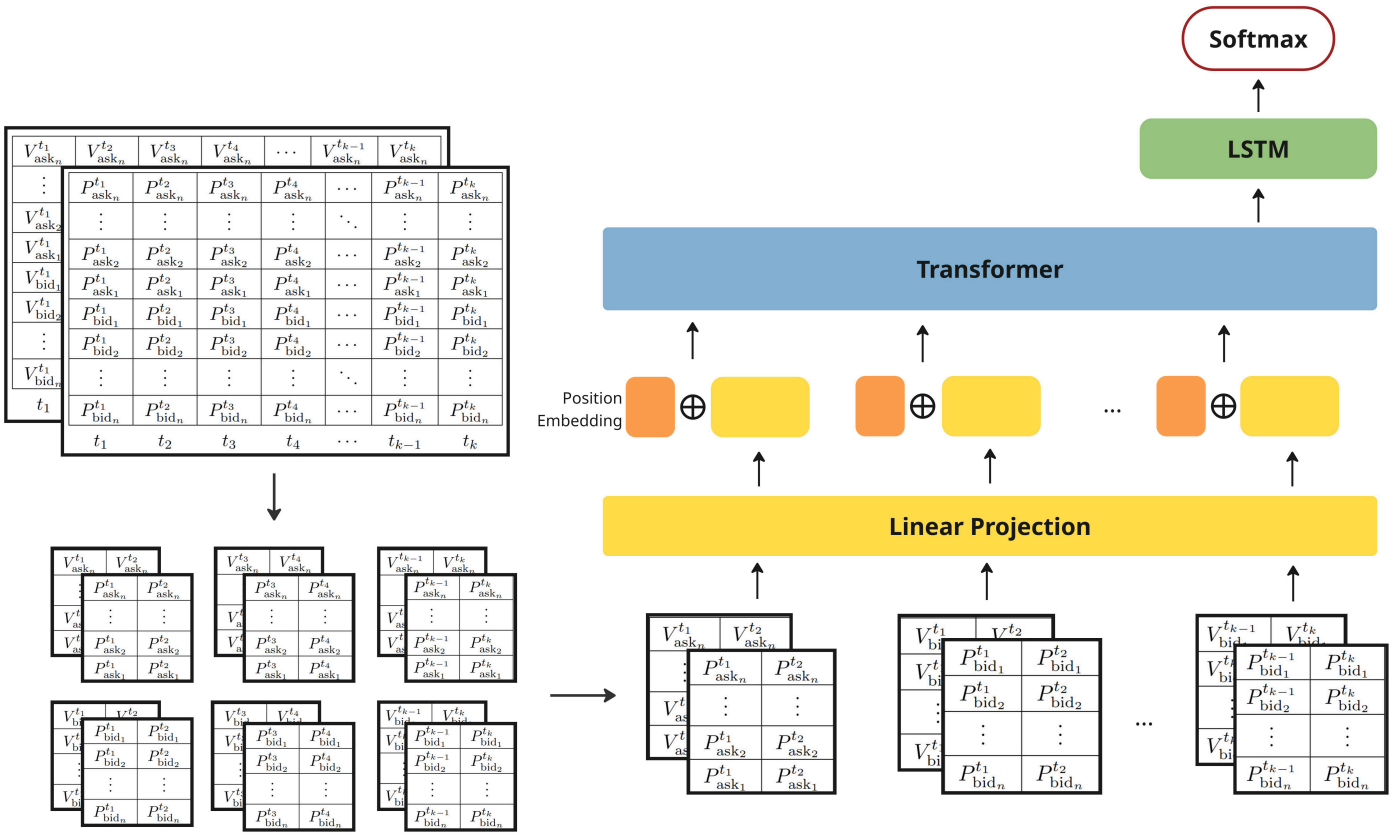
LiT model architecture.

In this section we discuss the proposed LiT model architecture (Figure 2) which consists of three main components: (1) a linear projection concatenated with positional embeddings to efficiently represent structured patches from the limit order book data; (2) transformer layers utilizing self-attention mechanisms to encode spatial and temporal dependencies between patches; and (3) LSTM layers to further model long-term temporal dependencies. Additionally, we provide details regarding the experimental training and fine-tuning settings.

### 4.1 LiT model architecture

#### 4.1.1 Input layers

Drawing inspiration from multi-channel representations in image processing (e.g., RGB channels), the grid-like structure of limit order book data is represented using two input channels: one for price and one for volume information. This results in a three-dimensional input **x**∈ℝ^*H*×*W*×*C*^ (Figure 2), where *H* denotes the depth of the LOB (i.e., the number of price levels), *W* is the window size representing the number of time steps used to construct each training example, and *C* is the number of channels which in this case is 2 for price and volume channels.

Following (Dosovitskiy et al., 2020), we split the input LOB data into patches to facilitate efficient feature extraction. However, as random small square patches in the LOB do not consistently represent coherent or interpretable market structures, they may span across sides or unrelated price levels without preserving meaningful spatial or temporal context, instead of sampling small square patches in random locations as done in image processing with resolution (*P, P*), where *P* is much smaller than both the height and width of the original image. In our approach, we adopt a structured patching scheme with size (*P*_*h*_, *P*_*w*_), where *P*_*h*_ denotes the vertical dimension of the patch, which is set equal to the number of price and volume levels in bid or ask side, i.e. *P*_*h*_ = *H*/2, and *P*_*w*_ is a small temporal window representing multiple ticks. This design ensures that each patch captures consistent and interpretable information across price levels while maintaining temporal locality. This results in a total number of *HW*/(*P*_*h*_×*P*_*w*_) patches extracted.

To retain information about position and structure, we incorporate a learnable positional embedding that encodes both side information and the temporal position of each patch. This embedding is concatenated with the linear projection of each patch and passed to the transformer layers for further encoding.

#### 4.1.2 Transformer layers

Inspired by how humans selectively focus on relevant information when processing complex data, the attention mechanism in deep neural networks was originally introduced by Bahdanau (2014) and popularized by Luong (2015) with several efficient variants. In this work, the transformer layers in our Limit Order Book Transformer model adopt the self-attention mechanism proposed by Vaswani et al. (2017). This mechanism effectively captures both spatial and temporal dependencies in LOB sequences by leveraging the importance of different patches based on their contextual relationships, while convolutional layers rely on fixed-sized filters and primarily capture local patterns. Furthermore, this mechanism also easily maintains efficiency by parallel processing sequence elements.

Specifically, self-attention computes attention scores using three vector representations derived from the input price and volume information: queries (**Q**), keys (**K**) and values (**V**). Given a query vector **q** and a key vector **k**_*j*_ (*j* = 1, …, *T*), the attention score is defined as:


$$\text{score}\left(\text{q},{\text{k}}_{j}\right)=\frac{{\text{q}}^{⊤}{\text{k}}_{j}}{\sqrt{d_{k}}}$$

where *d*_*k*_ is the dimensionality of the vectors. These scores quantify the relevance of each patch in the context of predicting future market movements. The attention weights, representing the normalized importance of each patch, are calculated as:


$$\alpha_{j}=\frac{\exp\left(\text{score}\left(\text{q},{\text{k}}_{j}\right)\right)}{\overset{T}{\underset{i=1}{\sum }}\exp\left(\text{score}\left(\text{q},{\text{k}}_{i}\right)\right)}$$

Subsequently, the context vector can be calculated with a weighted sum over the value vectors:


$${\text{c}}^{\text{att}}=\overset{T}{\underset{j=1}{\sum }}\alpha_{j}{\text{v}}_{j}$$

This is then passed through a feedforward network to produce the final transformer layer output **z**_*t*_.

#### 4.1.3 LSTM and output layers

For the market movement forecasting task, the transformer layer final output **z**_*t*_ is then fed to the LSTM layers to further model temporal dependencies within the encoded LOB features. A The LSTM layers enhance the capture of sequential patterns that complement the self-attention mechanism by explicitly maintaining temporal states, which from a high level the LSTM is given by


$$\left({\text{h}}_{t},{\text{c}}_{t}\right)=\text{LSTM}\left({\text{z}}_{t},{\text{h}}_{t-1},{\text{c}}_{t-1}\right)$$

where **h**_*t*_ denotes the hidden state, **c**_*t*_ the memory cell state, **h**_*t*−1_, **c**_*t*−1_ the recurrent states from the previous timestep. Finally, the output layer applies softmax to classify the market trends.

### 4.2 Training and fine-tuning settings

For all our experiments, the models are implemented in Python using Keras (Chollet, 2015) and TensorFlow (Abadi et al., 2015) and all the models are implemented with a comparable number of parameters. All the training and evaluation are performed on King's Computational Research, Engineering and Technology Environment (CREATE) (King's College London, 2025). The adaptive optimisation method RMSProp was employed during training, and each model was trained for up to 300 epochs, with early stopping applied after 30 epochs without improvements to ensure convergence, and a batch size of 128 was used throughout the experiments. During the training stage for the experiments in Section 5.2, all parameters are learnable. In the fine-tuning phase for the experiments in Section 5.4, all layers are frozen except for the last two fully connected layers, allowing the model to adapt to the data distribution of the latest market conditions.

## 5 Results

### 5.1 Experiment setup

In all experiments, we use the 64 most recent snapshots as the input to our model. To ensure numerical stability during the training process, we apply *z*-score normalization separately for volume and price data. Furthermore, to verify the robustness and adaptability of our model across different prediction horizons, we calculate price changes and define market trends over four time windows: 300 ms–500 ms, 300 ms–700 ms, 300 ms–1,000 ms and 500 ms–1,000 ms, we calculate the average price change and exclude timestamps where no change in the mid-price occurs. This results in an unevenly spaced time series of market movement events. The mid-price change is defined as:


$$\text{price\_change}=\frac{\frac{1}{k}\overset{t+k}{\underset{i=t+1}{\sum }}p_{mid}^{i}-P_{mid}^{t}}{P_{mid}^{t}}$$

Where k denotes the number of future timesteps within the time window. To classify the market trends, we use a specific percentage *p* of the current price as a threshold $p\times P_{mid}^{t}$ to group the market movements into three following categories:

- *upward*: the price is increasing and the price change is over *p* percentage of the previous price.
- *stable*: the price change is within *p* percentage of the previous price.
- *downward*: the price is decreasing and the price change is over *p* percentage of the previous price.

And the following metrics are assessed across all the experiments.


(1) $$\begin{array}{lll} Accuracy & = & \frac{TP+TN}{TP+TN+FP+FN} \end{array}$$

(2) $$\begin{array}{lll} Precision & = & \frac{TP}{TP+FP} \end{array}$$

(3) $$\begin{array}{lll} Recall & = & \frac{TP}{TP+FN} \end{array}$$

(4) $$\begin{array}{lll} F1 & = & \frac{2*Precision*Recall}{Precision+Recall}=\frac{2*TP}{2*TP+FP+FN} \end{array}$$

Due to potential class imbalance introduced by the choice of different thresholds (in our experiments, we select 0.000015 to maintain a relatively balanced label distribution), we normalize the evaluation metrics based on class frequencies. Specifically, for a classification task with *C* classes with the sizes for each class (*n*_*i*_, *i* = 1, 2, .., *C*), for class *i*, we then rescale its metrics by weight *W*_*i*_:


$$W_{i}=\frac{\sum n_{i}}{C*n_{i}}$$

### 5.2 Comparison of forecasting models

**Figure 3:**
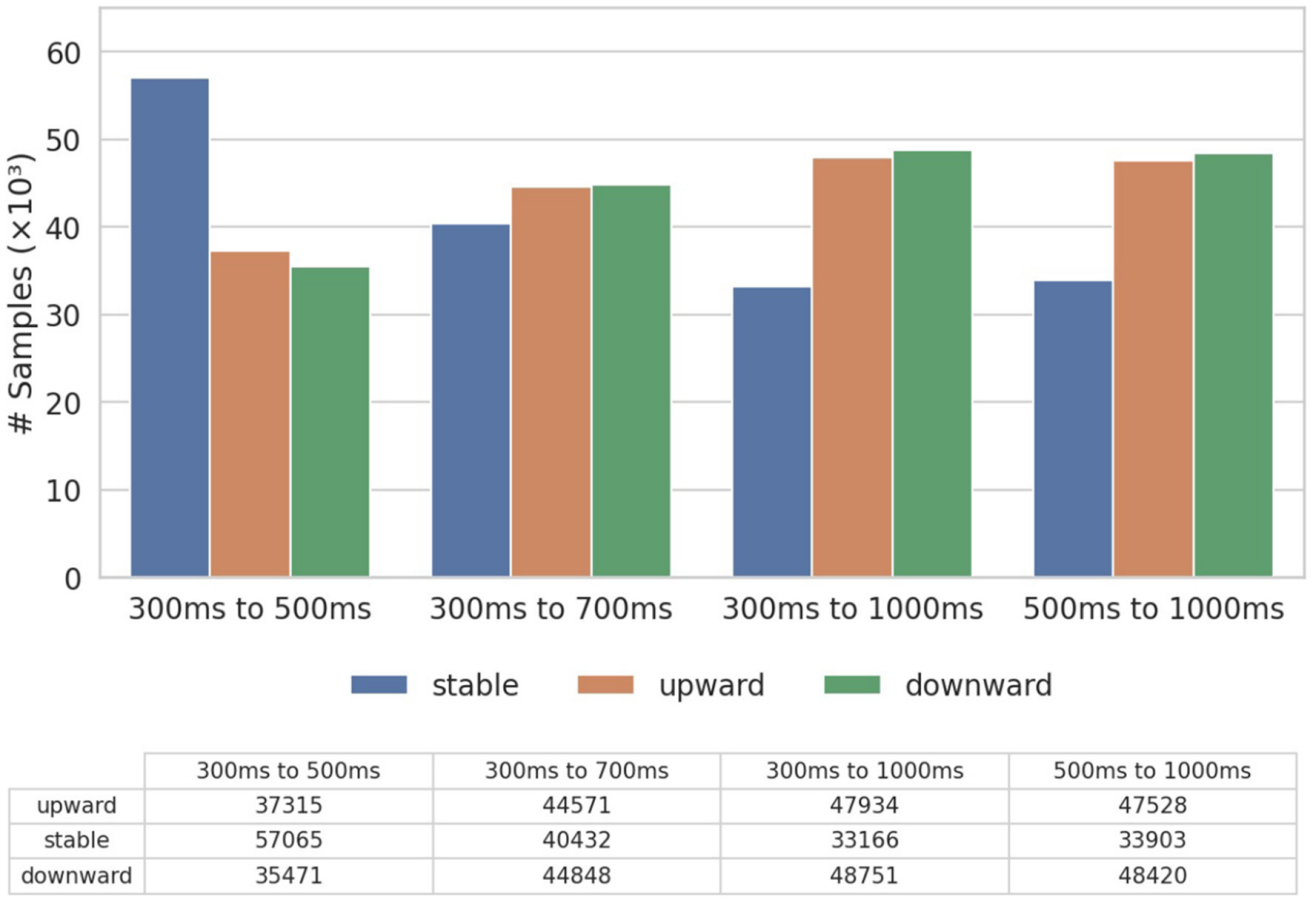
Distribution of training labels.

In this section, we evaluate the performance of our proposed model (LiT) against a range of traditional ML models and deep learning baselines. Specifically, for the traditional models, we include Ridge Regression (RR), Random Forest (RF) and Support Vector Machine (SVM), while the deep learning baselines consist of Multilayer Perceptron (MLP) and Long Short-Term Memory (LSTM) (Hochreiter and Schmidhuber, 1997). Additionally, we compare LiT with the vanilla transformer structure (ViT) used in vision tasks (Dosovitskiy et al., 2020) and two state-of-the-art models with convolution layers designed for limit order book data: DeepLOB (Zhang et al., 2019) and TransLOB (Wallbridge, 2020). For this comparison, due to the computational constraints and scalability limitations of certain baseline models, we use a subset of the entire September dataset and the label distribution is shown in Figure 3. We show the assessment of the transfer learning capability by pre-training on the full dataset and fine-tuning on a future dataset in the next Section. To assess the robustness of our model across different horizons, we evaluate it using all four time windows in a time-series cross-validation setup. Specifically, the dataset is split into five folds, and we report the mean of all evaluation metrics across these folds. We show that even without relying on convolutional layers, LiT consistently outperforms both traditional and deep learning baselines, as well as existing state-of-the-art models, demonstrating its ability to capture microstructural market dynamics effectively.

Table 2 presents the forecasting results for the shortest prediction horizon of 300 ms to 500 ms, which evaluates the ability to capture immediate microstructural dynamics. The results show that, although model performance is under 60% in all metrics, highlighting the challenge due to the noise and volatility inherent in ultra-short-term price movements, deep learning models MLP and LSTM generally show improvements of 1–3% over traditional ML models, and the state-of-the-art models further improve upon the deep learning baselines by an additional 1–2%. Among state-of-the-art models, the ViT model lags behind other models, suggesting that the vanilla vision-based transformers without an LSTM head are less effective at modeling LOB data. Despite DeepLOB achieving the highest precision, its relatively lower recall leads to a weaker F1 score and accuracy compared to TransLOB and LiT. In the forecasting for this prediction horizon, both LiT and TransLOB outperform DeepLOB, with LiT achieving the highest F1 score (58.99%) and accuracy (59.03%), indicating a marginal but meaningful advantage in this short-term forecasting window.

**Table 2:** Market movement forecasting results: 300 ms–500 ms horizon.

**Model**	**Precision (%)**	**Recall (%)**	**F1 score (%)**	**Accuracy (%)**
SVM	55.00	56.50	54.25	56.40
RR	59.37	53.99	47.32	53.99
RF	57.25	58.50	56.75	58.66
MLP	56.51	56.21	56.15	56.21
LSTM	57.91	57.31	57.14	57.31
ViT	56.90	56.89	54.87	56.89
DeepLOB	**59.65**	57.66	57.09	57.66
TransLOB	59.14	58.84	58.77	58.84
LiT	59.10	**59.02**	**58.99**	**59.03**

Bold values indicate the best (highest) result for each metric.

Table 3 shows the forecasting results for the 300 ms–700 ms window, a slightly longer and more stable prediction horizon. The results show that, with the increased time window, all models demonstrate improved performance, with most metrics surpassing 60%, indicating greater predictability over longer intervals. Regarding the comparison across models, as in the previous setting, deep learning models outperform traditional approaches by a 1–3% margin while state-of-the-art models outperform deep learning baselines by 1–3%. DeepLOB and TransLOB perform similarly, while the ViT model again underperforms, and LiT achieves the best overall results in F1 Score (63.65%) and Accuracy (64.58%), suggesting stronger generalization in capturing medium-horizon trends.

**Table 3:** Market movement forecasting results: 300 ms–700 ms horizon.

**Model**	**Precision (%)**	**Recall (%)**	**F1 score (%)**	**Accuracy (%)**
SVM	58.00	58.75	58.00	58.77
RR	**64.21**	60.80	60.10	60.80
RF	62.50	62.75	62.25	62.91
MLP	60.73	62.71	60.54	62.71
LSTM	61.86	63.17	61.89	63.17
ViT	59.69	59.14	59.37	59.14
DeepLOB	63.26	63.98	63.20	63.98
TransLOB	63.12	64.43	63.05	64.43
LiT	63.49	**64.59**	**63.65**	**64.58**

Bold values indicate the best (highest) result for each metric.

Table 4 shows the results for the longest forecasting window of 300 ms to 1,000 ms. With model performance close to 70% across several metrics, we observe a consistent improvement due to the increased temporal aggregation of market dynamics, which helps mitigate the short-term noise and volatility. Among the models, the state-of-the-art models, except ViT, which shows only moderate improvement and continues to underperform relative to others, once again achieve the best forecasting results, while traditional ML models continue to show the weakest performance. We observe LiT achieves the best results across all four metrics—Precision (66.20%), Recall (68.34%), F1 Score (66.40%), and Accuracy (68.34%)—demonstrating its ability to capture longer-term dependencies in market dynamics effectively. Notably, DeepLOB achieves a comparable F1 Score to LiT but a lag in Accuracy, while conversely, TransLOB produces a close Accuracy but a noticeably lower F1 Score, suggesting LiT offers a better balance in all metrics, resulting in more stable and reliable performance.

**Table 4:** Market movement forecasting results: 300 ms–1,000 ms horizon.

**Model**	**Precision (%)**	**Recall (%)**	**F1 score (%)**	**Accuracy (%)**
SVM	59.75	61.50	58.75	61.64
RR	65.22	62.52	59.18	62.52
RF	64.25	65.50	62.75	65.37
MLP	62.04	65.16	61.83	65.16
LSTM	65.77	67.64	66.10	67.63
ViT	62.75	63.79	63.15	63.79
DeepLOB	65.92	67.76	66.23	67.76
TransLOB	65.83	68.25	65.68	68.25
LiT	**66.20**	**68.34**	**66.40**	**68.34**

Bold values indicate the best (highest) result for each metric.

Table 5 reports the results for a shifted prediction window starting from 500 ms to 1,000 ms. This allows us to validate model robustness over slightly delayed inputs and different temporal offsets. Results remain consistent with earlier findings. LiT again performs competitively, with the highest recall (66.37%) and accuracy (66.37%) and near-best F1 score. While DeepLOB slightly edges out LiT in F1, the overall margin is minimal, affirming LiT's stable and competitive performance across time horizons.

**Table 5:** Market movement forecasting results: 500 ms–1,000 ms horizon.

**Model**	**Precision (%)**	**Recall (%)**	**F1 score (%)**	**Accuracy (%)**
SVM	58.50	60.00	57.75	60.07
RR	63.89	60.71	57.68	60.71
RF	62.50	63.50	61.50	63.54
MLP	59.99	61.68	60.60	61.68
LSTM	62.16	64.68	62.14	64.68
ViT	60.81	61.40	61.07	61.40
DeepLOB	**64.27**	65.99	**64.57**	65.99
TransLOB	63.63	66.24	63.49	66.24
LiT	64.14	**66.37**	64.32	**66.37**

Bold values indicate the best (highest) result for each metric.

### 5.3 Comparison of different patch sizes

To examine how the structured patching scheme in LiT influences model performance, we evaluate different combinations of patch height (**H**), width (**W**), and the resulting number of patches (**N**) with the same dataset as in Section 5.2. These settings correspond to how the input LOB training example is partitioned into structured patches before being passed to the transformer layers. As illustrated in Figure 2, the input LOB data represented as a grid of price and volume information is divided into vertically and horizontally aligned rectangular patches. The structured patching preserves both spatial structure across price levels and temporal structure across different timestamps. Specifically, in our comparison of different patch sizes:

- **H** represents the patch height corresponding to spatial depth (i.e., the number of LOB price levels).
- **W** represents the patch width corresponding to the temporal window (i.e., the number of timestamps).
- **N** represents the resulting number of patches.

Table 6 shows the forecasting results for the 300 ms–500 ms horizon across different patch sizes. For a fixed patch height (**H**), we observe a consistent pattern across all settings that narrower temporal widths (**W**) lead to better performance. Specifically, patches with **W** = 4 outperform **W** = 8 by 1–2%, which in turn outperform **W** = 16 by a comparable margin. This suggests that a higher temporal resolution within each patch more effectively captures the microstructural dynamics in LOB data. Conversely, while fixing the patch width and comparing different depths (**H**), performance generally improves with increasing patch height, suggesting that capturing a greater LOB depth helps to capture underlying market dynamics. Overall, the configuration (**H** = 20, **W** = 4) achieves the best results for this short-term forecasting horizon.

**Table 6:** Forecasting results for different patch sizes: 300 ms–500 ms horizon.

**H**	**W**	**N**	**Precision (%)**	**Recall (%)**	**F1 score (%)**	**Accuracy (%)**
10	4	64	**59.30**	58.89	58.74	58.89
10	8	32	57.05	56.97	56.93	56.97
10	16	16	55.99	55.95	55.91	55.95
20	4	32	59.24	**59.06**	**59.00**	**59.07**
20	8	16	57.74	57.52	57.44	57.52
20	16	8	56.16	56.06	56.02	56.06
40	4	16	59.10	59.02	58.99	59.03
40	8	8	57.78	57.51	57.43	57.51
40	16	4	55.77	55.60	55.54	55.60

Bold values indicate the best (highest) result for each metric.

Across Tables 7–9, we observe similar trends that performance improves consistently with narrower temporal windows and deeper spatial windows. Unlike the more marginal improvements observed in the 300 ms–500 ms horizon, these longer horizons demonstrate a clearer benefit from having a greater window height (**H**) and narrower window width (**W**). In particular, the configuration (**H** = 40, **W** = 4) consistently achieves the best results across all metrics and horizon time windows, highlighting the importance of both spatial depth and high-frequency temporal resolution when modeling LOB dynamics over extended periods.

**Table 7:** Forecasting results for different patch sizes: 300 ms–700 ms horizon.

**H**	**W**	**N**	**Precision (%)**	**Recall (%)**	**F1 score (%)**	**Accuracy (%)**
10	4	64	61.66	63.09	61.71	63.08
10	8	32	60.97	62.94	60.73	62.94
10	16	16	60.17	62.27	60.11	62.27
20	4	32	63.39	64.53	63.54	64.53
20	8	16	61.60	63.02	61.83	63.02
20	16	8	60.11	62.06	60.12	62.06
40	4	16	**63.49**	**64.59**	**63.65**	**64.58**
40	8	8	61.92	63.32	62.04	63.32
40	16	4	59.84	62.01	59.74	62.01

Bold values indicate the best (highest) result for each metric.

**Table 8:** Forecasting results for different patch sizes: 300 ms–1,000 ms horizon.

**H**	**W**	**N**	**Precision (%)**	**Recall (%)**	**F1 score (%)**	**Accuracy (%)**
10	4	64	64.95	67.50	65.10	67.50
10	8	32	63.95	66.89	64.22	66.89
10	16	16	63.46	66.62	63.55	66.62
20	4	32	66.00	68.03	66.28	68.03
20	8	16	64.48	67.23	64.70	67.23
20	16	8	63.38	66.54	63.47	66.54
40	4	16	**66.20**	**68.34**	**66.40**	**68.34**
40	8	8	65.13	67.53	65.38	67.53
40	16	4	63.36	66.62	63.15	66.62

Bold values indicate the best (highest) result for each metric.

**Table 9:** Forecasting results for different patch sizes: 500 ms–1,000 ms horizon.

**H**	**W**	**N**	**Precision (%)**	**Recall (%)**	**F1 score (%)**	**Accuracy (%)**
10	4	64	63.02	65.72	63.02	65.72
10	8	32	61.87	65.03	61.88	65.03
10	16	16	61.23	64.58	61.27	64.58
20	4	32	63.94	66.11	64.31	66.11
20	8	16	62.08	65.04	62.25	65.04
20	16	8	61.05	64.45	61.08	64.45
40	4	16	**64.09**	**66.37**	**64.32**	**66.37**
40	8	8	62.72	65.26	62.95	65.26
40	16	4	60.95	64.52	60.64	64.52

Bold values indicate the best (highest) result for each metric.

Overall, the evaluation metrics across four prediction horizons show clear sensitivity to patch size. Across all horizons, narrower temporal windows (**W** = 4) with greater spatial depth (**H** = 40) consistently yield stronger results. While larger patch sizes reduce the cost of computation resources, this trade-off comes at the compromise of predictive performance. These results suggest that LiT benefits most from a patching strategy that balances spatial coverage with high temporal granularity, enabling transformer layers to effectively attend to high-frequency structural changes within the LOB.

### 5.4 Adaptation across time via fine-tuning

In the context of rapidly evolving market dynamics and high-volume streaming data, fully retraining a model to keep up with the current market state is often computationally and operationally impractical. Moreover, for limit order forecasting tasks, when there is a shift in data distribution between the training and deployment periods, model performance tends to degrade over time (Fang et al., 2021), indicating that models trained on historical data alone may not remain effective in evolving market conditions. While the primary goal of this paper is to assess the performance of the proposed model in capturing market microstructure dynamics compared to other methods, we also explore how LiT can be pre-trained on historical data and fine-tuned for market states in the subsequent periods and help mitigate this challenge. In this section, we first illustrate how model trained on a static historical dataset performs over time, and then show how transfer learning through pre-training and fine-tuning can help maintain its practical capability to adapt to changing market conditions. Specifically, we show how LiT can be pre-trained on a large historical dataset and then fine-tuned on more recent data, enabling the model to quickly adjust to new market states without full retraining.

We use all datasets described in Section 3 in this experiment. Specifically, the entire September data is used to pre-train a large model. For the October, November and December datasets, we apply a simple 60/40 split for the training and testing sets instead of cross-validation. In order to verify the distribution shift in the LOB data and assess the model adaptability, we compare three training strategies:

- **From-scratch**: for each of the October, November, and December datasets, a model is trained solely on its training set and evaluated on its test set. These results serve as the baseline for comparison.
- **Zero-shot**: a model is first pre-trained on the September data and then evaluated on the test sets of October, November and December. This setup helps assess the impact of distributional shift without adaptation.
- **Fine-tuning**: for each of the October, November, and December datasets, the same pre-trained September model is first fine-tuned on its training set by freezing all layers except for the final dense layers, then evaluated on its test set. This setup demonstrates the benefit of adaptation to recent market conditions.

Table 10 presents the results for each month using the 300 ms–500 ms forecasting horizon (similar trends observed across other horizons). For the From-scratch approach, where the model is trained on the most recent data, the results remain relatively stable across all months, with all metrics consistently around 62–63%. For the Zero-shot approach, where the model pre-trained on September data is applied directly to the test sets of future months without adaptation, we observe an obvious degradation in performance over time. Specifically, while October results remain strong due to the recency and size of the September dataset, we observe a significant decline of around 5% in most metrics in November and December, falling even below the from-scratch baselines. This confirms the presence of a distribution shift between the September training data and the test set of target months, and indicates the limitations of applying static trained models in evolving market conditions. In contrast, the Fine-tuned model demonstrates clear improvements. By adapting a pre-trained model to the most recent market conditions, it outperforms both Zero-shot and From-scratch approaches and consistently achieves the best performance across all evaluation metrics for all four months. The gains are especially notable in November and December, where fine-tuning recovers the performance lost in the Zero-shot setting and exceeds From-scratch baselines. These findings highlight the effectiveness of fine-tuning LiT in adapting to shifting market dynamics. They also demonstrate that the earlier layers in LiT successfully learn robust and transferable representations of LOB features, allowing adaptation to new market conditions through fine-tuning only the final layers. Combined with its non-convolutional design, scalability, and fast fine-tuning capability, LiT offers a highly practical solution for real-time deployment in dynamic financial markets.

**Table 10:** Monthly forecasting results.

**Model**	**Precision (%)**			**Recall (%)**			**F1 score (%)**			**Accuracy (%)**		
	**Oct**	**Nov**	**Dec**	**Oct**	**Nov**	**Dec**	**Oct**	**Nov**	**Dec**	**Oct**	**Nov**	**Dec**
From-scratch	62.47	62.09	63.93	62.34	62.09	63.87	62.12	62.04	63.86	62.34	62.09	63.87
Zero-shot	64.20	63.89	60.77	64.19	59.57	59.32	64.17	58.04	58.14	64.19	59.57	59.32
Fine-tuned	**65.26**	**64.71**	**64.33**	**65.07**	**64.66**	**64.12**	**64.88**	**64.58**	**64.13**	**65.07**	**64.66**	**64.12**

Bold values indicate the best (highest) result for each metric.

## 6 Conclusion

This paper introduced LiT (Limit Order Book Transformer), a transformer-based model designed to capture microstructural dynamics in high-frequency financial markets without relying on convolutional layers. Unlike prior approaches that rely heavily on convolutional layers, LiT leverages a vision-inspired transformer-based architecture to effectively model both spatial and temporal dependencies in LOB sequences.

We evaluated LiT on datasets collected from the Binance exchange and compared it against traditional machine learning models, early deep learning architectures and state-of-the-art LOB models such as DeepLOB and TransLOB. Through extensive experiments across multiple forecasting horizons, we demonstrated that LiT consistently outperforms all baselines in precision, recall, F1 score, and accuracy, showing its capability to learn fine-grained LOB features without the need for CNN-based feature extraction.

We also investigated how different patch configurations affect the performance of LiT. Across all forecasting horizons, we found that using narrower temporal windows and deeper spatial windows significantly improves performance. These results confirm the importance of patch configurations in transformer-based LOB models and provide practical insights for designing effective architectures to capture high-frequency market microstructural dynamics.

Beyond static evaluation, we further explored the adaptability of LiT in dynamic market conditions. By pre-training the model on historical data and fine-tuning on more recent periods, we showed that LiT can effectively adjust to shifting market dynamics. Our results showed that zero-shot transfer leads to performance degradation due to distributional shift, while fine-tuning not only helps mitigate this issue but also surpasses from-scratch baselines. This demonstrates LiT's practical value in real-world scenarios, where full retraining is often computationally infeasible and rapid adaptation is essential. Its ability to combine transformer-based sequence modeling with efficient fine-tuning makes it particularly well-suited for modern financial environments, where models must not only learn complex patterns but also remain robust in the face of constant market evolution.

While LiT demonstrates strong forecasting performance and market adaptability, there are several promising directions for future work. Currently, the model relies solely on raw price and volume data. Incorporating additional high-frequency features such as order imbalance could potentially further enhance predictive performance. Another potential direction is to extend LiT within a reinforcement learning framework, enabling it not only to forecast price movements but also to learn optimal trading strategies through interaction with a market environment.

## Data Availability

The original contributions presented in the study are included in the article/supplementary material, further inquiries can be directed to the corresponding author.
